# How corals get their nutrients

**DOI:** 10.7554/eLife.90916

**Published:** 2023-08-18

**Authors:** Elizabeth A Hambleton

**Affiliations:** 1 Division of Microbial Ecology, Centre for Microbiology and Environmental Systems Science, University of Vienna Vienna Austria

**Keywords:** algae, Symbiodiniaceae, symbiosis, cell wall, monosaccharide, coral, Other

## Abstract

Algae living inside corals provide sugars for their host by digesting their own cell walls.

**Related research article** Ishii Y, Ishii H, Kuroha T, Yokoyama R, Deguchi R, Nishitani K, Minagawa J, Kawata M, Takahashi S, Maruyama S. 2023. Environmental pH signals the release of monosaccharides from cell wall in coral symbiotic alga. *eLife* **12**:e80628. doi: 10.7554/eLife.80628.

Living inside corals are a family of algae known as Symbiodiniaceae, which provide nutrients that corals need to survive (LaJeunesse et al., 2018). However, rising water temperatures are putting corals under stress, causing them to eject their algae and lose their color. Dramatic images of this heat-driven coral reef ‘bleaching’ have drawn attention to the breakdown of the symbiotic relationship between coral animals and their algae. With entire ecosystems relying on this close-knit relationship, it is important to understand how corals and algae interact and how this might be altered by changing environmental conditions.

The algae reside within an acidic compartment inside coral cells where they can perform photosynthesis (Barott et al., 2015). They use sunlight to fix carbon from the environment and transfer the resulting energy-rich nutrients – such as lipids, amino acids and sugars – to their coral hosts (Rosset et al., 2021; Figure 1A). Glucose is one of the key sugars transferred (Burriesci et al., 2012), which coral cells then take up using specialized transporters, such as those in the GLUT family (Sproles et al., 2018). But how the sugars get out of algae in the first place is largely unknown. Previous studies have suggested that algae may release glucose using transporter proteins that respond to sugar concentrations in the environment (Xiang et al., 2018). Now, in eLife, Shinichiro Maruyama (Tohoku University and Ochanomizu University) and colleagues from multiple institutes in Japan – including Yuu Ishii as first author – report that algae can also secrete sugars by degrading their cell walls (Ishii et al., 2023).

**Figure 1.**
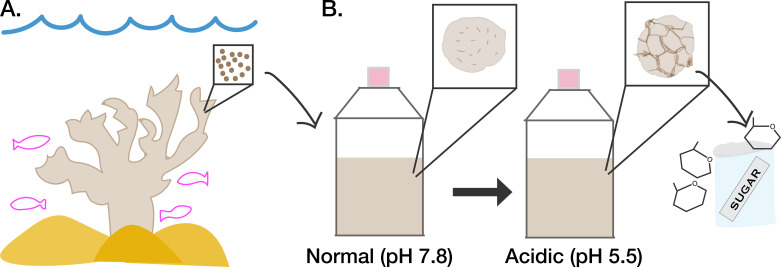
Acidic conditions affect how symbiotic algae secrete sugar. (**A**) A family of algae known as Symbiodiniaceae (represented as brown circles in inset) live within reef-building corals (beige) where they transfer nutrients they have fixed via photosynthesis to their hosts. (**B**) The symbiotic algae can also grow in culture under normal conditions (pH 7.8, left), in which they display a smooth appearance (depicted in left inset). When the culture medium of the algae is made more acidic (pH 5.5) to mimic the internal environment of coral (right), the algae display a more wrinkled cell surface (depicted in right inset). The wrinkled cell surface is due to the algae digesting their own cell walls using activated cellulase enzymes. This results in increased secretion of sugars into the surrounding environment that can then be taken up by coral cells.

The team studied a species of algae from the Symbiodiniaceae family known as *Breviolum minutum* which were living freely outside of a host. To recreate the environment algae experience inside coral cells, the pH of the medium the algae were cultured in was changed from normal sea water levels (pH 7.8) to a more acidic formula (pH 5.5). This decreased the rate at which algae grew and photosynthesized, which led Ishii et al. to believe that nutrient transfer would also be inhibited. Instead, they found that the algae were actually secreting more glucose as well as another sugar called galactose. Chemically blocking photosynthesis with a targeted inhibitor also increased sugar output, contradicting previous expectations that this process is required for nutrient transfer (Burriesci et al., 2012).

To explain these findings, Ishii et al. used scanning electron microscopy to take detailed images of the surfaces of the algae, which have been shown to respond to glucose in the environment (Xiang et al., 2018). This revealed that under acidic conditions, algae surfaces changed from mostly smooth to mostly wrinkled (Figure 1B). Interestingly, the wrinkle pattern appeared to correspond to the sites where the plates that make up the algae cell wall join together (LaJeunesse et al., 2018), suggesting that acid affects these weaker junctions between plates first. Transmission electron scanning microscopy showed that the interior organization of the algae was also disrupted, with the cell wall pulling away from the body of the cell.

To understand how acidity increases sugar output and cell wall degradation, Ishii et al. compared the genes algae switched on when cultured in normal versus acidic conditions. Out of the genes that were only switched on in acidic conditions, several encoded proteins which help to break down carbohydrates. These included the genes for cellulases, enzymes that digest cellulose, which is a key component of the cell wall in algae and plants. Bringing these threads together, Ishii et al. blocked cellulase activity with a targeted drug, and found that this decreased the algae’s glucose and galactose outputs.

The results reveal a pathway by which symbiotic algae degrade their cell walls to release key nutrients, which can then be taken up by their coral host. Importantly, Ishii et al. point out that a combination of both digestion- and transporter-driven secretion are likely involved in sugar output. Transporter-driven secretion has been suggested to take around an hour and to rely on photosynthesis (Burriesci et al., 2012). Meanwhile, the digestion method discovered by Ishii et al. is slower (one day) and independent of sunlight, suggesting that it may mobilize stored materials when photosynthesis is not possible (e.g. in darkness). The symbiotic system may therefore be tunable according to the dynamic conditions of the coral reef.

While culture conditions of free-living algae may not always perfectly recreate the complex interior of a host cell (Maruyama and Weis, 2021), these results likely also apply to algae living in coral. Indeed, Ishii et al. found published datasets showing that algae cultured in normal (non-acidic) conditions regulate a key cellulase gene differently compared to symbiotic algae, supporting their finding that these cellulases are activated by acidic environments including those in coral cells (Xiang et al., 2020).

Future studies could examine the sugar export mechanisms of other species of symbiotic algae, particularly those which have been shown to influence the cellular acidity and bleaching tolerance of their coral hosts (Allen-Waller and Barott, 2023). As ocean temperatures continue to rise, these kinds of investigations are more important than ever.
